# Validation of LRG1 as a Potential Biomarker for Detection of Epithelial Ovarian Cancer by a Blinded Study

**DOI:** 10.1371/journal.pone.0121112

**Published:** 2015-03-23

**Authors:** Jing Wu, Haidi Yin, Jianhui Zhu, Ronald J. Buckanovich, Jason D. Thorpe, Jianliang Dai, Nicole Urban, David M. Lubman

**Affiliations:** 1 University of Michigan, Department of Surgery, Ann Arbor, MI, United States of America; 2 University of Michigan, Department of Internal Medicine, Ann Arbor, MI, United States of America; 3 Fred Hutchinson Cancer Research Center, Seattle, WA, United States of America; 4 University of Texas MD Anderson Cancer Center, Biostatistics, Houston, TX, United States of America; Queen Mary Hospital, HONG KONG

## Abstract

**Background:**

Leucine-rich alpha-2-glycoprotein (LRG1) was found to be differentially expressed in sera from patients with Epithelial Ovarian Cancer (EOC). The aim of this study is to investigate the performance of LRG1 for detection of EOC, including early stage EOC, and to evaluate if LRG1 can complement CA125 in order to improve EOC detection using two independent blinded sample sets.

**Methods and Results:**

Serum LRG1 and CA125 were measured by immunoassays. All assays were performed blinded to clinical data. Using the two independent sample sets (156 participants for sample set 1, and 233 for sample set 2), LRG1 was differentially expressed in EOC cases as compared to healthy, surgical, and benign controls, and its performance was not affected by the conditions of blood collection. The areas under the ROC curve (AUC) for LRG1 in differentiating EOC cases from non-cases were 0.797 and 0.786 for sample set 1 and 2. For differentiating EOC cases from healthy controls, the AUC values for LRG1 were 0.792 and 0.794. At a fixed specificity of 95%, LRG1 detects 52%, and 53.5% of EOC cases from healthy controls for sample set 1 and 2. When combining LRG1 and CA125, the AUC value increased to 0.927, which was improved compared to CA125 (AUC=0.916) (*p*=0.008) alone in distinguishing EOC cases from non-cases. More importantly, LRG1 also showed potential performance in differentiating early stage EOC from non-cases with an AUC of 0.715 for sample set 1, and 0.690 for sample set 2. The combination of LRG1 and CA125 resulted in an AUC of 0.838, which outperforms CA125 (AUC=0.785) (*p*=0.018) in detecting early stage EOC cases from non-cases using the larger sample set.

**Conclusions:**

LRG1 could be a useful biomarker alone or in combination with CA125 for the diagnosis of ovarian cancer.

## Introduction

With an estimated 21,980 new cases of ovarian cancer and 14,270 deaths in 2014, ovarian cancer ranks as the fifth leading cause of cancer death among women in the United States [1]. The increased mortality associated with ovarian cancer relates primarily to the lack of disease-specific symptoms, especially for early stage ovarian cancer. The 5-year survival rate for patients with advanced ovarian cancer (stage III/IV) is only 15%-20%, while the cure rate for early stage disease (stage I/II) can approach >90% [2]. Therefore, diagnostic markers for early stage ovarian cancer have the potential to improve the overall survival rate for this disease.

Serum CA125 identified in human ovarian carcinoma cell lines [3], is the most widely used serum biomarker for detection of ovarian cancer. While elevated levels of CA125 are frequently observed in advanced stage ovarian cancer, a number of limitations of CA125 used as the diagnostic or prognostic marker for ovarian cancer have been demonstrated in various studies [4]: CA125 is elevated in less than 50% of early stage ovarian cancers [5], CA125 may be elevated due to a number of benign and malignant conditions unrelated to ovarian cancer [6], and while the elevated levels of CA125 are strongly associated with serous tumors, it is less sensitive to the non-serous histologies [7].

Currently, the most often used screening modalities for the detection of ovarian cancer are transvaginal ultrasounds (TVS) and the CA125 serum marker level [8]. However, the recently published results from the PLCO trial which included 34,261 healthy women, failed to show a mortality reduction with annual screening with CA125 and TVS and reported a substantial increase in the use of invasive medical procedures some of which resulted in unnecessary morbidities [9–10]. Thus, there is a critical need to identify additional noninvasive biomarkers that complement CA125 in order to achieve improved performance in detecting ovarian cancer.

Numerous studies have been carried out to discover serum biomarker candidates for ovarian cancer detection using mass spectrometry-based proteomics, enzyme-linked immunosorbent assay (ELISA), gene/protein expression microarrays, and multiplex bead-based immunoassays. Several promising candidate biomarkers including human epididymis protein 4 (HE4) [11], mesothelin [12], apolipoprotein A1 (Apo A1) [13], epidermal growth factor receptor [14], and transferrin [15] have been discovered in these studies. From these markers and others, a number of promising multimarker panels have been recently developed, which showed improved sensitivity and specificity to differentiate early stage ovarian cancer as compared to CA125 alone [16–18]. However, most of these biomarkers or multimarker panels have not yet been shown to reduce mortality, and these biomarkers showed poor performance when validated in pre-diagnostic samples [19]. Therefore, better biomarkers are needed in order to reduce mortality from ovarian cancer through early detection.

In our previous work, we identified a panel of glycoprotein biomarkers for detection of EOC using an integrated platform containing mass spectrometry and lectin microarray, and LRG1 was found to be differentially expressed in early stage EOC cases [20–21]. Recently, through peptidome analysis of urine from 6 ovarian cancer patients and 6 healthy controls, Smith *et al*.[22] found the high abundance of LRG1 peptides in all of the samples from ovarian cancer patients, and only one peptide from one healthy control, indicating LRG1 may serve as potential urine biomarker for detection of ovarian cancer after further validation using larger numbers of urine samples.

In the present study, we sought to validate the performance of LRG1 in differentiating EOC, including early stage EOC from healthy controls and non-cases, and to test if LRG1 can complement CA125 in order to improve EOC detection using two independent blinded sample sets, one of which included paired specimens collected at the time of surgery and in the clinic prior to surgery to identify possible biases introduced by systematic differences in the conditions of blood collection between cases and controls [23].

## Materials and Methods

### Materials

ELISA kit for LRG1 was purchased from IBL International (Hamburg, Germany). CA125 ELISA kit was purchased from Genway (San Diego, CA, USA).

### Patient Population and Study Design

Specimens for this study are provided by the Pacific Ovarian Cancer Research Consortium/SPORE in Ovarian Cancer (POCRC, www.pocrc.org) [24]. The POCRC maintains a repository of biological specimens from women with and without ovarian cancer for research use. All patient recruitment and enrollment, specimen collection and specimen processing are conducted through the POCRC. These activities have been reviewed by the FHCRC Institutional Review Board and are approved under FHCRC IR file numbers #4771 and #4563. This study is also approved by the University of Michigan (IRB00005467).

Two serum banks were used here. The first sample set for the preliminary verification included 156 serum specimens from 35 apparently healthy controls (healthy women, cancer-free), 16 controls from women undergoing surgery for benign, non-ovary-related conditions such as uterine fibroids (surgical controls), 43 from women undergoing surgery for benign ovarian disease such as a benign serous cystadenoma or endometriosis (benign controls), 20 stage I/II EOC cases, and 42 stage III/IV EOC cases. To assess whether individual markers are affected by the conditions of the blood draw, the sample set 1 includes paired specimens collected 1 or more days prior to surgery (pre-surgical specimens) and on the day of surgery after anesthesia was administered and before the surgical procedure (surgical specimens) from 33 individuals, including 19 cases, 13 benign controls, and 2 surgical controls. Among the EOC cases, the pre-surgical specimens were collected between 2 and 18 days before surgery (median: 7 days). Each control group was age matched to the cases, and as a result, subject age is not associated with case/control status (*p*>0.05, Table 1).

**Table 1:** Age and marker values for the 156 participants from the blinded sample set I.

	Healthy control	Surgical control^a^	Benign control	EOC cases	EOC cases	
	(n = 35)	(n = 16)	(n = 43)	(n = 62)	I/II (n = 20)	III/ /IV (n = 42)
Age, median (range)^*b*^,y	57 (43–86)	52 (30–71)	65 (37–81)	57 (20–88)	54 (39–84)	57 (20–88)
LRG1, median (range)^*c*^, μg/mL	15.9 (7.1–48.0)	11.6 (7.9–28.2)	16.3 (3.1–73.9)	24.9 (5.7–93.3)	19.1 (13.0–45.2)	31.0 (5.7–93.3)
(10^th^, 90^th^ percentile)	(9.5–26.1)	(8.2–23.7)	(9.2–27.7)	(13.1–66.1)	(13.9–34.4)	(13.0–75.0)
CA125, median (range)^*d*^, U/mL	21.8 (5.6–978.6)	22.3 (10.6–31.8)	22.4 (7.2–79.6)	262.2 (10.2–3172.0)	84.5 (10.2–484.4)	579.1 (24.8–3172.0)
(10^th^, 90^th^ percentile)	(8.3–52.6)	(10.9–29.6)	(9.1–61.0)	(25.7–1602.0)	(16.0–465.5)	(63.6–1973.0)

^a^Surgical control: specimens collected from women undergoing surgery for benign, non-ovary-related conditions such as uterine fibroids.

^b^ *p*>0.05 for difference among all stages.

^c^ *p*<0.05 for differences between stage III/ /IV and controls and stage I/II and controls.

^d^ *p*<0.05for differences between stage III/ /IV and controls and stage I/II and controls.

The second sample set, a larger blinded confirmation set, is composed of 233 patients including 77 healthy controls, 32 surgical controls, 53 benign diseases, 27 stage I/II EOC cases, and 44 stage III/IV EOC cases. Specimens were randomized onto three plates for ELISA assays. All lab work was performed blinded and the data from sample set 1 was provided to the investigators only after laboratory work was completed. Sample set 2 was never unblinded. Statistical analyses were performed by a statistician from the POCRC (J.D.T).

### ELISA Assays

The ELISA assays were performed according to the manufacturer’s instructions. The absorbance values were read at a wavelength of 450 nm. The calibration curves were constructed using purified protein standards. The concentrations of proteins assessed were calculated from their specific calibration curves.

### Statistical Analysis

Nonparametric Wilcoxon tests and Dunn’s multiple comparisons were used to determine whether CA125, LRG1, and participant age differed between healthy controls, surgical controls, benign disease, and EOC and between early stage and late stage EOC. For all statistical comparisons, *p*<0.05 was taken as statistically significant.

Retrospective power analyses were conducted for a given sample size, variability of biomarker expression and the observed differences. For a two-tailed test at the 0.05 significance level, the power to identify systematic differences in the expression of LRG1, and CA125 were greater than 99%, which provides the statistical support for the number of samples included in our study.

To evaluate the performance of these markers, the receiver operating characteristic (ROC) curves were constructed and the area under the ROC curve (AUC) and the corresponding 95% confidence intervals (CI) were used to evaluate the overall performances of the markers. Multivariate analysis was done by logistic regression to find the best-fitting multivariate model for each comparison group. The maximum of sensitivity (SE) and specificity (SP) that reflects the intention to maximize the correct classification rate was used as an optimal standard to find the best cutoff values [25], where the corresponding sensitivity and specificity were calculated under these cutoff values. The AUC accuracy for the ROC curve of the combined analysis was checked by 10-fold cross-validation.

## Results

### Determination of Serum Concentrations of Biomarkers in Patients with EOC by ELISA Assays

In previous work, we found the abnormal expression of LRG1 in ovarian cancer, including in early stage cases [20]. Herein, we further validated our findings using two independent blinded sample sets to evaluate the performance of LRG1 or in conjunction with CA125 in detecting ovarian cancer. Serum specimens were randomized prior to shipping to the testing laboratory and the assays were performed blinded to case status.

**Fig 1:**
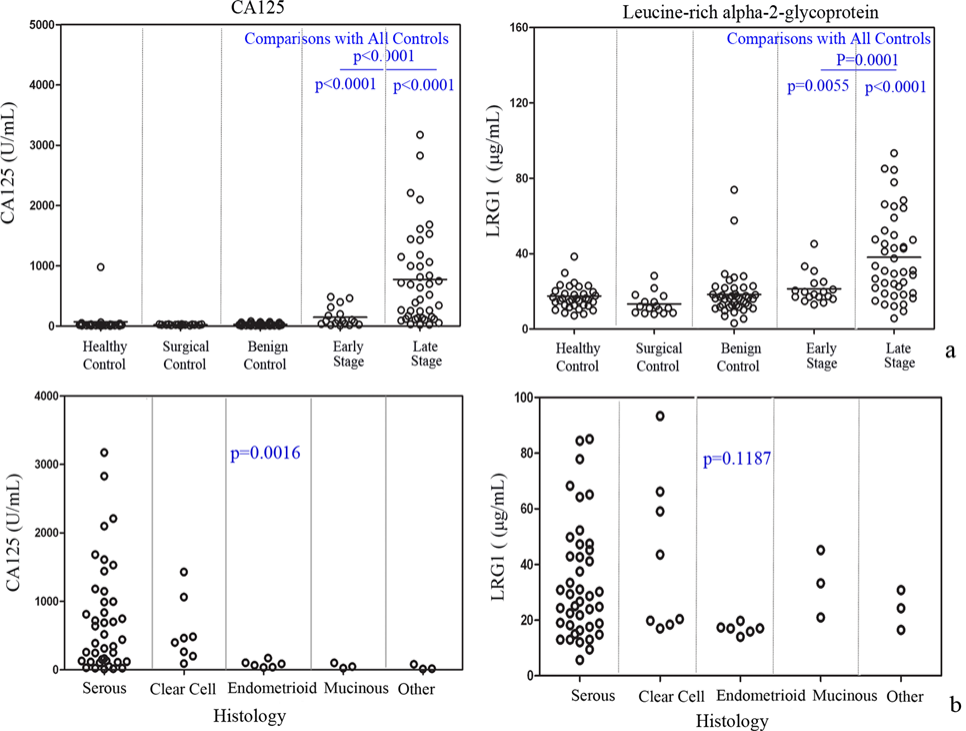
Serum marker levels for sample set 1. (a) The levels of CA125, and LRG1 were examined in the serum from healthy controls, surgical controls, benign diseases, stage I/II EOC, and stage III /IV EOC. *p<0.05 indicates a significant difference between pairwise comparisons (all cases vs. all controls; early stage cases vs. all controls; late stage cases vs. all controls). (b) Marker distributions for the EOC patients from sample set 1 with different histological subtypes.

The distribution of marker values by EOC cases and non-cases in 156 serum samples from the preliminary confirmation set 1 is shown in Table 1. LRG1 and CA125 are elevated relative to control groups in both early and late stage EOC cases (*p*<0.01 for all pairwise comparisons) as shown in Fig. 1a. In addition, CA125 expression differed significantly among the histological subtypes (*p* = 0.002, Fig. 1b), while LRG1 did not differ among different subtypes of EOC (*p*>0.05).

Neither LRG1 nor CA125 were found to differ significantly between pre-surgical and surgical samples, indicating LRG1 and CA125 concentrations were not affected by the conditions of blood collection (Figure S1 in S1 File). LRG1 and CA125 both showed good performance in differentiating cases from controls regardless of whether specimens were drawn at surgery or prior to surgery (Figure S2 in S1 File).

**Fig 2:**
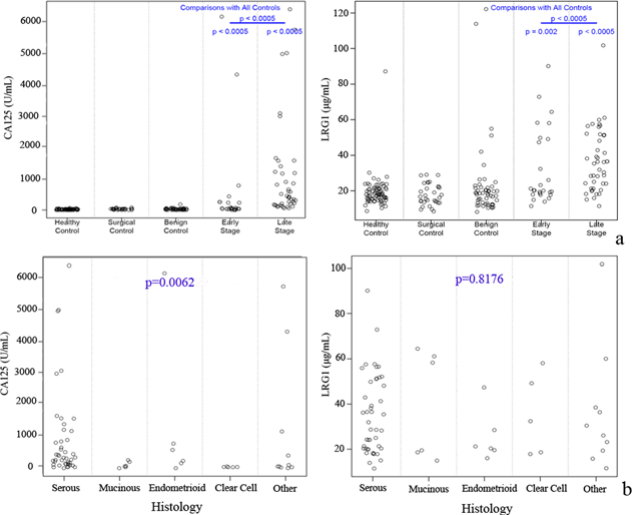
Serum marker levels for sample set 2. (a) The protein level changes of CA125 and LRG1 were confirmed in the larger sample set 2. (b) Marker distributions for the EOC patients with different histological subtypes.

The differential expression of LRG1 between cases and non-cases was further validated using an independent larger blinded sample set 2, and consistent with the results using sample set 1, LRG1 was elevated in EOC cases, including both early- and late- stage cases as compared to non-cases (Fig. 2a). Similar results showing that CA125 is significantly differentially expressed among the various histologies was observed in sample set 2, and LRG1 was not found to differ by histology (*p*>0.05, Fig. 2b).

The concentrations of LRG1 and CA125, and the clinical characteristics for each participant from sample set 1 and sample set 2 are shown in S1 Table and S2 Table.

### ROC Performance Analysis for the Candidate Markers to Differentiate EOC cases from Non-Cases

**Fig 3:**
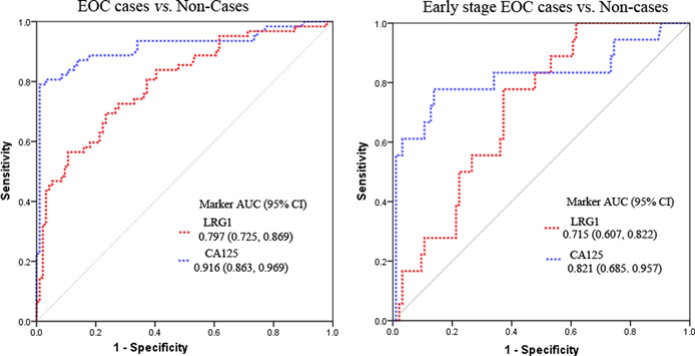
ROC curves comparing marker concentrations in cases to non-cases for sample set 1. (a) ROC analyses for CA125 and LRG1 to differentiate EOC from non-cases. (b) ROC analyses for CA125 and LRG1 to differentiate early stage EOC from non-cases.

ROC curves were constructed to compare the sensitivity and specificity of each marker for distinguishing between EOC cases, including early- and late-stage cases from non-cases (healthy, surgical, and benign controls) as shown in Fig. 3a. The AUCs for CA125 and LRG1 were 0.916, and 0.797, respectively. Using a cutoff that maximizes sensitivity+specificity, CA125 provided a sensitivity of 79% and specificity of 98% at a cut-off of 81 U/mL, while LRG1 showed a sensitivity of 70% and specificity of 76% at a cut-off of 18.75 μg/mL to differentiate EOC cases from non-cases. At 95% specificity, the sensitivities for CA125 and LRG1 were 80.6% and 46.8%, respectively.

To distinguish early stage EOC from non-cases, CA125 had an AUC of 0.821 and LRG1 had an AUC of 0.715 (Fig. 3b). By the optimal cutoff that maximizes the sensitivity+specificity, CA125 provided a sensitivity of 78% and specificity of 86% at a cut-off of 34.4 U/mL, while LRG1 showed a sensitivity of 78% and specificity of 63% at a cut-off of 17 μg/mL to differentiate early stage EOC from non-cases.

Performance of candidate markers in differentiating EOC from non-cases was further confirmed using sample set 2. Consistent with the results from sample set 1, the AUCs for CA125 and LRG1 were 0.915, and 0.786 for differentiating EOC cases from non-cases (Figure S3 in S1 File). For distinguishing early EOC cases from non-cases, the AUCs for CA125 and LRG1 were 0.785, and 0.690 using the sample set 2 (Figure S4 in S1 File).

### ROC Performance of the Candidate Markers for Differentiating EOC Cases from Healthy Controls

**Fig 4:**
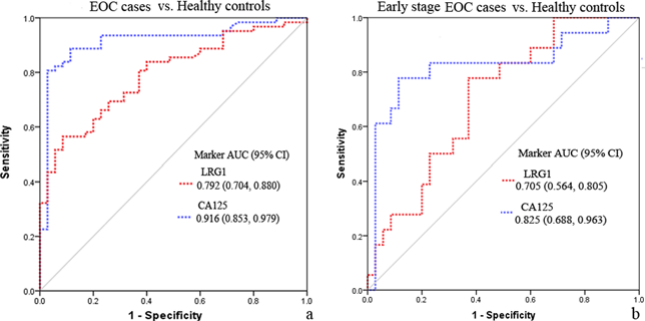
ROC curves comparing marker concentrations in cases to healthy controls for sample set 1. (a) ROC analyses for CA125 and LRG1 to differentiate EOC from healthy controls. (b) ROC analyses for CA125 and LRG1 to differentiate early stage EOC from healthy controls.

The AUC values for CA125, and LRG1 to distinguish EOC from healthy controls were 0.916, and 0.792, respectively (Fig. 4a). By the optimal cutoff that maximizes the sensitivity+specificity, CA125 provided a sensitivity of 90% and specificity of 89% at a cut-off of 30.9 U/mL, while LRG1 showed a sensitivity of 57% and specificity of 91% at a cut-off of 23.64 μg/mL to differentiate EOC from healthy controls. At a fixed specificity of 95%, CA125 detects 82.3%, and LRG1 detects 52% of EOC cases (Fig. 4a). To differentiate early stage EOC from healthy controls, the AUC values for CA125, and LRG1 were 0.825, and 0.705 (Fig. 4b).

The performance of LRG1 for discriminating EOC from healthy controls was also confirmed in the larger blinded sample set 2. The AUC values for CA125 and LRG1were 0.938 and 0.794, respectively, and at a 5% false-positive rate, CA125 detects 80.3%, and LRG1 detects 53.5% of EOC cases (Figure S5 in S1 File).

### Multimarker Panel Analysis

**Fig 5:**
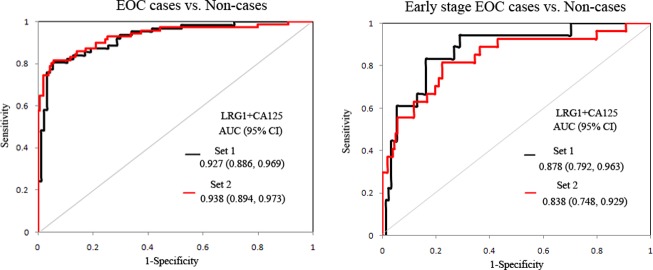
Multimarker panel analysis. The performance of multimarker panels in distinguishing EOC/ early stage EOC from non-cases using sample set 1and sample set 2.

Multivariate logistic regression was used to identify optimal coefficients for combining CA125 and LRG1 in a marker panel. The combination of LRG1 and CA125 had an AUC of 0.927 (OR_LRG1_ = 1.053, *p* = 0.016; OR_CA125_ = 1.011, *p* = 0.001), which outperforms CA125 (AUC = 0.916) (*p* = 0.008, LR test) in distinguishing EOC from non-cases (Fig. 5), indicating that LRG1 may provide independent diagnostic value in addition to CA125. Using the larger sample set 2, a similar result was obtained where the combination of LRG1 and CA125 had an AUC of 0.933 (OR_LRG1_ = 1.062, *p* = 0.013; OR_CA125_ = 1.361, *p*<0.001), which outperforms CA125 (AUC = 0.915) (*p* = 0.009, LR test) in distinguishing EOC from non-cases. Furthermore, to distinguish early stage EOC from non-cases, the combination of LRG1 and CA125 resulted in an AUC of 0.838 (OR_LRG1_ = 1.058, *p* = 0.018; OR_CA125_ = 1.211, *p* = 0.001), which was improved compared to CA125 (AUC = 0.785) (*p* = 0.018, LR test), as shown in Fig. 5.

## Discussion

CA125 is the most promising serologic biomarker for preoperative evaluation of patients with pelvic masses, for follow-up of patients after treatment, and to assess the response to chemotherapy of ovarian cancer [8]. However, CA125 is not an effective screening tool where its levels are elevated in only 50 to 60% of women with early stage ovarian cancer and a number of benign conditions can cause elevation of CA125 levels [26]. Therefore, we sought to identify useful biomarkers or complementary markers to CA125 to improve its performance in diagnosis of EOC, especially in detecting early stage EOC.

Human LRG1 is a serum glycoprotein with five potential glycosylation sites, and has been found to be elevated in pancreatic cancer [27] and lung cancer [28]. However, there are few reports about LRG1 in detecting EOC, especially in detecting early stage EOC. In this study, using two independent sample sets we measured the protein level of LRG1 in the sera of EOC patients using ELISA assay, and evaluated the performance of LRG1 or in conjunction with CA125 in distinguishing EOC cases from non-cases.

Moreover, we introduced a sample set including paired pre-surgical and at surgical specimens in order to avoid the false validation result which may be caused by biases in conditions of blood collection. Using this sample set, we found LRG1 was not affected by the conditions of blood collection, and showed good performance to differentiate EOC from controls regardless of whether sera were collected at surgery (AUC = 0.784) or in a short interval prior to surgery (AUC = 0.839), as shown in Figure S2 in S1 File. To further exclude possible biases caused by conditions of surgery such as anesthesia, and stress [23], we also introduced surgical controls to the sample set 1 and sample set 2. With these two sample sets, LRG1 did not show significant changes between healthy controls and surgical controls (Figs. 1a and 2a), which also indicated that elevated LRG1 is specific to malignancy.

At present there are no individual candidate markers that can outperform CA125 in detecting ovarian cancer. Therefore, more studies have focused on developing mutlimarker panels that can improve the sensitivity and specificity in detecting ovarian cancer compared to CA125 alone [16, 29]. In our study, we found that the combination of CA125 and LRG1 showed improved performance to distinguish early stage EOC from non-cases compared to CA125 alone.

Interestingly, using both sample set1 and sample set 2, we found that LRG1 did not vary in expression among the investigated histopathologic subtypes. In contrast, CA125 differed significantly among those subtypes (*p*<0.01), consistent with previous studies reporting that elevated levels of CA125 are most strongly associated with serous tumors [7]. Although identification of subtype specific markers for diagnosis of ovarian cancer has been addressed in several studies [30], uniform markers that can detect all major cancer subtypes are essential in broad application screening.

## Conclusion

In summary, using two blinded independent sample sets, LRG1 was found to be a promising candidate marker for detection of EOC and it also showed the potential to become a useful biomarker for the diagnosis of early stage EOC. The combination of LRG1 and CA125 showed improved performance to distinguish EOC including early stage EOC from non-cases compared to CA125. The performance of LRG1 for detection of EOC, especially early stage EOC, as well as its performance across different histological subtypes needs to be further validated using a larger sample set from multiple study sites, and in preclinical samples obtained from asymptomatic women.

## Supporting Information

S1 File Combined file of supporting figures. Figure S1: LRG1 and CA125 levels classified by population and surgical status in sample set 1. Figure S2: ROC analyses for CA125 and LRG1 for differentiating EOC cases from non-cases by conditions of blood collection. Figure S3: ROC analyses for CA125, and LRG1 to differentiate EOC cases from non-cases using sample set 2. Figure S4: ROC analyses for CA125, and LRG1 to differentiate early EOC cases from non-cases using sample set 2. Figure S5: ROC analyses for CA125, and LRG1 to differentiate EOC cases from healthy controls using sample set 2.
(DOCX)

S1 Table Clinical characteristics and LRG1, CA125 levels for each participant from sample set 1. (XLSX)

S2 Table Clinical characteristics and LRG1, CA125 levels for each participant from sample set 2. (XLSX)
