# Supplementary material for: Validation of LRG1 as a Potential Biomarker for Detection of Epithelial Ovarian Cancer by a Blinded Study
Source: PLoS One. 2015 Mar 23;10(3):e0121112. doi: 10.1371/journal.pone.0121112 (PMC4370724; doi:10.1371/journal.pone.0121112)

**
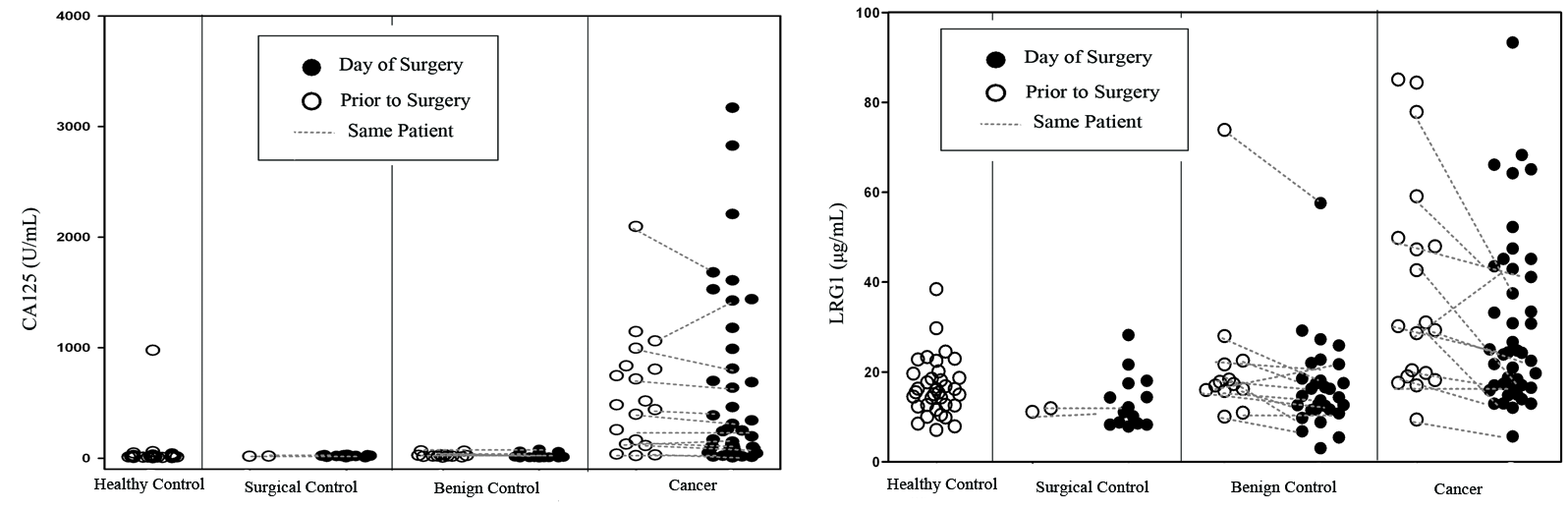
Supplemental Fig. S1**. **LRG1 and CA125 levels classified by population and surgical status in sample set 1.** Dotted lines connect the paired pre-surgical and surgical marker levels from the same patients.

**
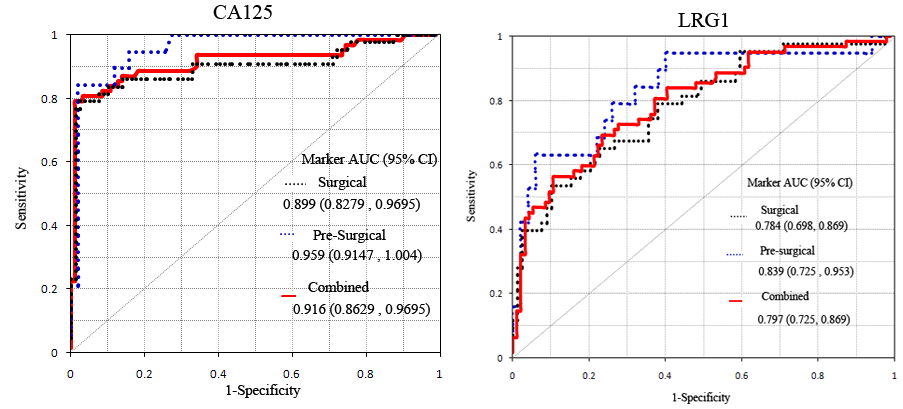
Supplemental Fig. S2**. **ROC analyses for CA125 and LRG1 for differentiating EOC cases from non-cases by conditions of blood collections.** Case specimens were collected at surgery (surgical) or 1to 18 days prior to surgery (pre-surgical).

**
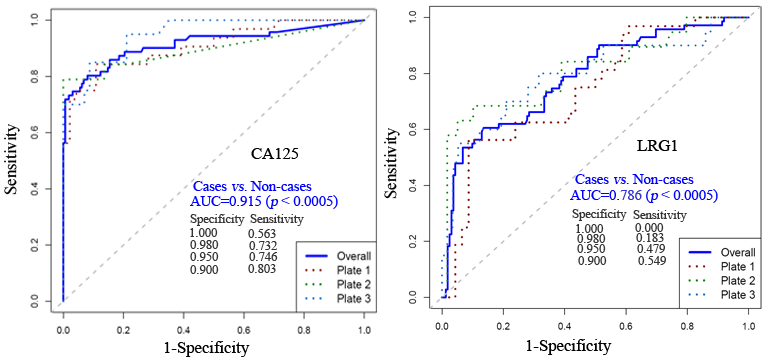
Supplemental Fig. S3**. **ROC analyses for CA125, and LRG1 to differentiate EOC cases from non-cases using sample set 2.**

**
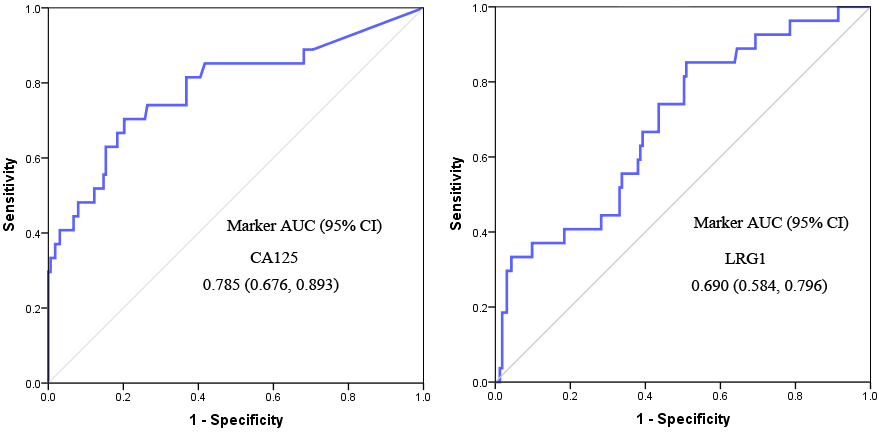
Supplemental Fig. S4**. **ROC analyses for CA125, and LRG1 to differentiate early stage EOC cases from non-cases using sample set 2.**

**Supplemental** **Fig. S5**. **ROC analyses for CA125, and LRG1 to differentiate EOC cases from healthy controls using sample set 2.**


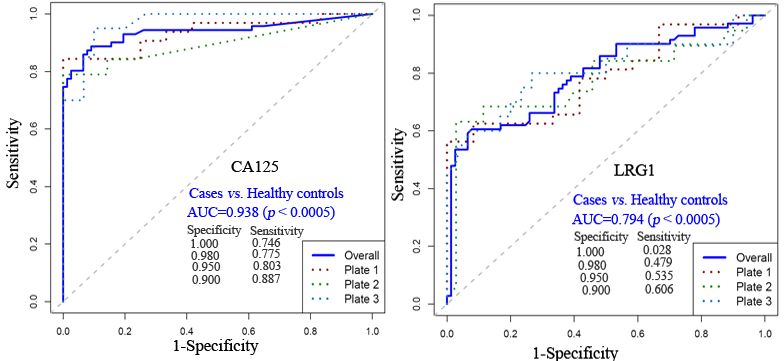

Supplement: S1 File — Figure S1: LRG1 and CA125 levels classified by population and surgical status in sample set 1. Figure S2: ROC analyses for CA125 and LRG1 for differentiating EOC cases from non-cases by conditions of blood collection. Figure S3: ROC analyses for CA125, and LRG1 to differentiate EOC cases from non-cases using sample set 2. Figure S4: ROC analyses for CA125, and LRG1 to differentiate early EOC cases from non-cases using sample set 2. Figure S5: ROC analyses for CA125, and LRG1 to differentiate EOC cases from healthy controls using sample set 2. (DOCX) [file pone.0121112.s001.docx]
